# T follicular helper cells expansion in transplant recipients correlates with graft infiltration and adverse outcomes

**DOI:** 10.3389/fimmu.2024.1275933

**Published:** 2024-02-07

**Authors:** Olivier Désy, Stéphanie Béland, Marie-Pier Thivierge, Meagan Marcoux, Jean-Simon Desgagnés, François Bouchard-Boivin, Alcino Gama, Julie Riopel, Eva Latulippe, Sacha A. De Serres

**Affiliations:** ^1^Transplantation Unit, Renal Division, Department of Medicine, University Health Center of Quebec, Faculty of Medicine, Laval University, Québec, QC, Canada; ^2^Pathology Division, Department of Medicine, University Health Center of Quebec, Faculty of Medicine, Laval University, Québec, QC, Canada

**Keywords:** kidney transplantation, follicular helper T cells, rejection, graft outcomes, anti-HLA antibodies

## Abstract

**Introduction:**

The process of immunization following vaccination in humans bears similarities to that of immunization with allografts. Whereas vaccination aims to elicit a rapid response, in the transplant recipient, immunosuppressants slow the immunization to alloantigens. The induction of CD4+CXCR5+ T follicular helper (Tfh) cells has been shown to correlate with the success of vaccine immunization.

**Method:**

We studied a cohort of 65 transplant recipients who underwent histological evaluation concurrent with PBMC isolation and follow-up sampling to investigate the phenotypic profiles in the blood and allotissue and analyze their association with clinical events.

**Results:**

The proportion of circulating Tfh cells was heterogeneous over time. Patients in whom this compartment increased had lower CCR7-PD1+CD4+CXCR5+ T cells during follow-up. These patients exhibited more alloreactive CD4+ T cells using HLA-DR-specific tetramers and a greater proportion of detectable circulating plasmablasts than the controls. Examination of baseline biopsies revealed that expansion of the circulating Tfh compartment did not follow prior intragraft leukocyte infiltration. However, multicolor immunofluorescence microscopy of the grafts showed a greater proportion of CXCR5+ T cells than in the controls. CD4+CXCR5+ cells were predominantly PD1+ and were in close contact with B cells in situ. Despite clinical stability at baseline, circulating Tfh expansion was associated with a higher risk of a composite of anti-HLA donor-specific antibodies, rejection, lower graft function, or graft loss.

**Conclusion:**

In otherwise stable patients post-transplant, circulating Tfh expansion can identify ongoing alloreactivity, detectable before allograft injury. Tfh expansion is relevant clinically because it predicts poor graft prognosis. These findings have implications for immune surveillance.

## Introduction

Allograft rejection is a process where the host becomes immune to graft antigens. Currently, the use of potent immunosuppressive agents reduces this immune reaction to the point where full-blown acute cellular rejection is infrequently observed. Nonetheless, signs of mild alloreactivity in the form of subtle leukocyte infiltration are frequently found when a graft is sampled (1). This process may be a prelude to a full-blown immune response leading to the development of allospecific B cells and antibody-mediated alloreactivity (1, 2). During chronic antibody-mediated rejection, the graft microcirculation is slowly obliterated by alloantibodies, which bind to the endothelium, resulting in relative hypoxia, fibrosis, and loss of function. When these immunological events are triggered, the host is ‘‘vaccinated’’ against the graft (3). No therapy exists that is successful or safe for reversing this process (3–7). Currently, this has been the leading cause of premature graft loss worldwide and the need for retransplants (8, 9).

Effectively tracking the sequence of immunological events upstream of developing high-affinity antibodies against grafts would be a major step in personalizing immunotherapy. Follicular helper T (Tfh) cells express CXCR5, which is the chemokine receptor that promotes their migration toward B cell follicles in the presence of the chemokine CXCL13 (10). Tfh cells are crucial for developing antibody responses by initially priming B cells at extrafollicular locations and in the germinal centers (GC) in the longer term (11, 12). In the non-transplant setting, the induction of CD4^+^CXCR5^+^ T cells correlates with immunization following vaccination, the capacity to respond to infections, and the development of autoimmune conditions (13–16). However, the chemokine receptors CXCR5 and CCR7 play mirror roles in the movement of T and B cells toward the T-B border zone of the lymphoid tissues in such a way that B cells progressively lose CXCR5 expression in favor of CCR7, while the opposite occurs in T cells, enabling both cell types to chemoattract to one another (11). IL-21, secreted by Tfh, is a key cytokine in the development of the humoral response by B lymphocytes (13). Elevated levels of circulating CD4^+^CXCR5^+^ T cells, which is a phenomenon commonly observed before symptomatic disease, have been reported in autoimmune diseases such as systemic lupus erythematosus, rheumatoid arthritis, and Sjogren’s syndrome (17). Similarly, chronic infections have been associated with persistent CD4^+^CXCR5^+^ T cell activation (18, 19). Tfh cells can also infiltrate inflamed tissues and in some cases form highly organized structures, including B lymphocytes and follicular dendritic cells, called ectopic lymphoid follicles or tertiary lymphoid structures. These structures can be found in situations of chronic inflammation, such as in autoimmune diseases, cancers and allograft rejection (20–22).

In the transplant setting, non-human primates transplanted following HLA sensitization as well as human kidney recipients with circulating anti-HLA antibodies, particularly those with antibodies directed against class II HLA, display increased CD4^+^CXCR5^+^ T cells in the blood (7, 23). However, these situations represent a late step in the alloimmunization process, in contrast to stable patients without evidence of circulating anti-HLA antibodies (24). We hypothesized that an increase in circulating CD4^+^CXCR5^+^ T cells in otherwise stable kidney recipients over time would indicate a subtle, ongoing alloimmune response, which could, in turn, lead to adverse graft outcomes. We show that some patients expand their circulating CD4^+^CXCR5^+^ T cells post-transplant. This increase was associated with lower surface expression of programmed cell death protein 1 (PD1) and ICOS over time, more abundant alloreactive CD4^+^ T cells, and higher levels of CD19^+^CD27^+^CD38^hi^ plasmablasts in the blood. Pathological examination of the allografts at baseline did not reveal ongoing rejection before an increase in CD4^+^CXCR5^+^ T cells. However, longitudinal follow-up revealed a more important loss of graft function and an increased risk of a composite of graft loss, donor-specific antibodies, rejection, and decline in function. Multicolor immunofluorescence microscopy identified a more important contingent of CXCR5^+^ T cells that were predominantly PD1-positive. Collectively, these results provide new insights into the dynamics and profiles of circulating CD4^+^CXCR5^+^ T cells post-transplant in humans.

## Materials and methods.

### Study design and clinical samples

We performed a single-center, observational cohort study based on PBMC samples collected between May 2016 and August 2018. Any kidney transplant recipient aged 18 years and above was eligible for enrollment in a prospective PBMC sample collection, biopsy section and clinical data biobank. Participation implied collection of clinical data and biological samples on the day of enrollment (time 0) and 3 months thereafter. Among the 152 consecutive patients enrolled during the period, we included all those who had undergone a biopsy confirming an absence of rejection at the time of enrollment. Overall, 65 patients meeting this criterion were included in the study. Samples included the first blood sample collected during graft biopsy and a follow-up sample at 3 months. The mean ± SD time post transplant to the first sample collection was 5.2 ± 6.4 years (**Table 1**).

**Table 1:** Demographics and baseline clinical characteristics.

CD4^+^CXCR5^+^ T_H_ M3/M0 ratio	≤0.75 (n=21)	0.76-1.53 (n=23)	≥1.53 (n=21)	P-value
Age (yr)	46 ± 15	57 ± 9	57 ± 14	0.01
Male gender	15 (71)	17 (74)	14 (67)	0.87
First transplant	13 (62)	15 (65)	19 (91)	0.08
Time post transplant (yr)	4.2 ± 5.2	5.3 ± 5.4	6.0 ± 7.5	0.86
eGFR at biopsy	53 ± 17	46 ± 14	48 ± 18	0.52
HLA A-B-DR-DQ mismatch				
HLA A mismatch	1.1 ± 0.7	1.2 ± 0.7	1.3 ± 0.7	0.77
HLA B mismatch	1.2 ± 0.7	1.2 ± 0.7	1.3 ± 0.7	0.99
HLA DR mismatch	0.6 ± 0.7	0.7 ± 0.8	0.7 ± 0.8	0.94
HLA DQ mismatch	0.9 ± 0.8	1.0 ± 0.7	0.8 ± 0.8	0.70
cPRA (%)	11 ± 26	22 ± 33	11 ± 20	0.31
0-19%	18 (85)	15 (65)	16 (76)	0.38
20-79%	2 (10)	6 (26)	5 (24)	
80-100%	1 (5)	2 (9)	0 (0)	
Donor type				0.57
Deceased	15 (71)	18 (82)	19 (91)	
Living - related	5 (24)	3 (14)	2 (9)	
Living - unrelated	1 (5)	1 (5)	0 (0)	
Induction				0.52
None	7 (34)	8 (35)	5 (24)	
Basiliximab	12 (57)	13 (57)	16 (76)	
ATG	2 (9)	2 (8)	0 (0)	
Maintenance immunosuppression				
Corticosteroids	26 (100)	25 (100)	26 (100)	–
Prednisone dose (mg)	7.7 ± 3.0	8.2 ± 2.4	7.8 ± 3.6	0.42
Tacrolimus	19 (91)	23 (100)	20 (95)	0.78
Tacrolimus T0 level (ng/dL)	6.2 ± 1.8	6.0 ± 1.8	6.3 ± 2.3	0.32
Mycophenolate	21 (100)	22 (96)	19 (91)	0.34
Mycophenolate dose (mg)^a^	1237 ± 395	1048 ± 368	1172 ± 416	0.29

Data are provided as mean ± standard deviation or n (%). Comparisons were performed using Kruskall-Wallis or Chi-square test. M3, month 3; M0, month 0; eGFR, estimated glomerular filtration rate, cPRA, calculated panel reactive antibodies.

a in mycophenolate mofetil equivalent.

### PMBC isolation, thawing, and cellular assay

PBMCs were isolated from heparinized blood using density gradient centrifugation (StemCell Technologies, Vancouver, Canada), washed two times with phosphate-buffered saline, resuspended in CTL-Cryo™ Media (Cellular Technology Limited, Shaker Heights, OH, USA), and stored in liquid nitrogen. For each patient, the baseline and follow-up samples were thawed on the same day through slow reconstitution with 1640 medium + 1% fetal bovine serum (Corning Cellgro, Manassas, VA, USA) and subsequently incubated in X-vivo 15 serum-free medium (Lonza, Walkersville, MD, USA) in duplicate in a 96-well plate at a concentration of 5 × 10^6^ cells/mL.

### Flow cytometry, antibodies, and reagents

The cells were stimulated for 5 h with anti-CD3/28 beads (Miltenyi Biotec, Auburn, CA, USA) and stained according to the manufacturer’s instructions. Cells were analyzed using an LSRFortessa cell analyzer (BD, Mississauga, ON, Canada). The gating strategy is displayed in **Supplementary Figure 1**. The following labeling antibodies were used: anti-CD4-VioBlue, anti-CXCR5-PE-Vio770, anti-ICOS-VioGreen, anti-CD19-VioBlue, anti-CD27-APC-Vio770, anti-CD38-FITC (Miltenyi), anti-PD1-FITC, and anti-CCR7-APC (BioLegend, San Diego, CA, USA). All antibodies were titrated against their respective isotypes. Flow cytometry data analyses were performed using the FlowJo vX software (FlowJo LLC). Absolute counts of CD4^+^CXCR5^+^ T cells in the peripheral blood were calculated by multiplying the percentages of CD4^+^CXCR5^+^ T cells measured by flow cytometry to the absolute counts of lymphocytes measured by the clinical hematology laboratory of the hospital using a blood sample drawn the same day.

### Tetramers

The following Class II tetramers were obtained from the National Institute of Health (NIH) Tetramer Core Facility, all PE-labeled: DRB1*01:01 human class II-associated invariant chain peptide (CLIP) 87-101, DRB1*04:01 human CLIP 87-101, and DRB1*15:01 human CLIP 87-101. The stock solutions were titrated and used at a final concentration of 6.5 μg/mL. In each experiment, at least 5 × 10^5^ cells were stained. Cells were incubated first with 50 nM Dasatinib (Sigma-Aldrich, Oakville, ON, Canada) per well for 30 min at 37°C. Subsequently, tetramers were added to the wells and incubated for 1 h at 4°C (25). Next, cells were stained for viability with efluor506 (Life Technologies, Carlsbad, CA, USA) as described above. To assess and subtract nonspecific binding, tetramers with an HLA allele unrelated to the donor were used as controls.

### Immunofluorescence staining of biopsy sections

Frozen sections were blocked and stained in two panels. In the first panel, the sections were stained with mouse anti-human CD3 and rabbit anti-human CXCR5 (Abcam, Toronto, ON, Canada) overnight at 4°C. After washing with phosphate-buffered saline, the sections were stained with secondary antibodies goat anti-mouse Alexa Fluor 594-conjugated and goat anti-rabbit Alexa Fluor 488-conjugated (Life Technologies) for 1 h at room temperature. In the second panel, they were stained with mouse anti-human CD279 (PD1, BioLegend) overnight, followed by goat anti-mouse Alexa Fluor 594-conjugated for 1 h at room temperature, and the following conjugated antibodies: anti-human CD19 Alexa Fluor 647 (BioLegend), anti-human CD4 Kirivia Blue 520 (BioLegend), and biotin anti-human CXCR5 (BioLegend). BV480 Streptavidin (BD) was used as the secondary antibody for CXCR5. Next, slides were mounted in a Slowfade Diamond antifade (Life Technologies). All sections were analyzed using a multicolor motorized fluorescence microscope IX83 (Olympus, Richmond Hill, ON, Canada) equipped with a dual color and monochrome CCD Olympus camera DP80, and images were analyzed using Olympus cellSens Dimensions Software.

### Pathological classification

The biopsies were assessed and graded according to the Banff working classification of renal allograft pathology (26–30). All biopsies were routinely presented during weekly transplant clinicopathological case reviews, during which both kidney pathologists reviewed challenging cases to ensure consistency in the evaluation.

### Anti-HLA antibody assessment

Anti-HLA antibody screening was routinely performed at 0, 1, 3, 6, and 12 months post-transplant and then annually as part of a routine clinical surveillance protocol. Anti-HLA antibody detection was also conducted at the time of biopsy or following any sensitizing events. None of the patients had donor specific antibodies at the time of transplantation. Serum samples were screened for *de novo* donor specific antibodies by flow cytometry using FlowPRA beads (One Lambda, Canoga Park, CA, USA). The Luminex Platform has been used to identify HLA antibodies using LABScreen single-antigen beads (One Lambda). A normalized mean fluorescence intensity (MFI) cut-off value of ≥ 1500 was used to detect dnDSA and their presence was confirmed in each case by the HLA laboratory director by eplet analysis.

### Statistics

Data were analyzed using the Wilcoxon signed-rank test, Mann–Whitney U test, Kruskal–Wallis test, or Spearman’s rank correlation. Multivariate linear regression and generalized linear models were used to adjust for clinical confounders. Survival analyses were conducted using the log-rank test and the proportional hazards model. The last observation (3^rd^ year) was carried forward to the 4^th^ year for estimated glomerular filtration rate (eGFR) when data were missing (n=12). All analyses were two-tailed, and a statistical significance was considered at P<0.05.

### Study approval

The study was approved by the Institutional Review Board of Quebec University Health Center (Project 2017-3606-F9-54172). Informed consent was obtained from all participants.

## Results

### An increase in circulating CD4^+^CXCR5^+^ T cells proportion over time is associated with the PD1^lo^CCR7^hi^ phenotype and lower ICOS expression

To characterize the evolution of the circulating Tfh cell population, we examined PBMC from 65 consecutive stable adult kidney recipients enrolled in a biobank, with an allograft biopsy confirming absence of rejection (**Table 1**). Blood samples were collected at the time of the biopsy (month 0) and three months later (month 3, **Table 1**).

**Figure 1:**
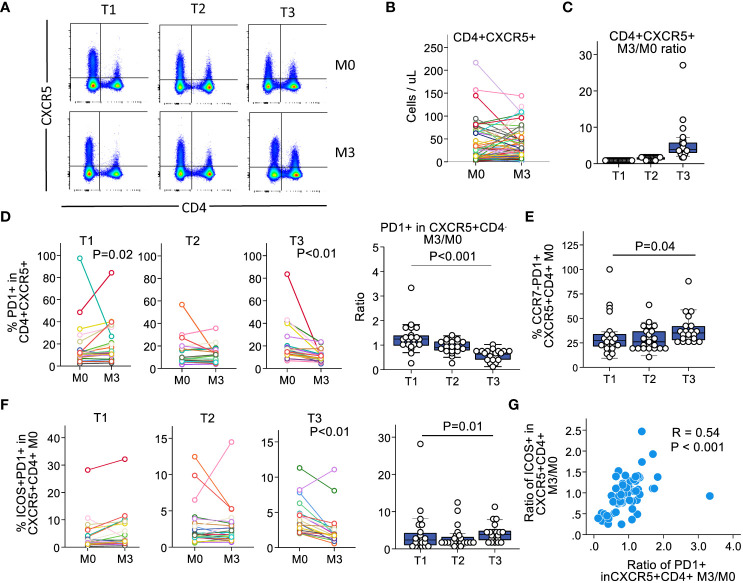
The heterogeneity in the circulating CD4+CXCR5+ pool over time correlates with variations in PD1 and ICOS expression. We classified the patients of the cohort according to the evolution in the expression of CXCR5+ in CD4+ T cells at baseline and at 3 months. **(A)** Representative examples of CXCR5 expression in CD4+ T cells across tertiles. **(B)** CD4+CXCR5+ counts in the blood. Each individual is illustrated as a single line. **(C)** Ratios of month 3 versus month 0 according to tertiles. **(D)** Expression of PD1 in CD4+CXCR5+ T cells for each individual by tertile (left) and summary ratios across tertiles (right). **(E)** Proportion of CCR7-PD1+ in CD4+CXCR5+ T cells across tertiles. **(F)** Proportion of ICOS+PD1+ in CD4+CXCR5+ T cells across tertiles. Left, comparison of M0 versus M3 for each individual illustrated as a single line, by tertile. Right, comparison between tertiles at M0. **(G)** Dotplots depicting the relationship between CD4+CXCR5+PD1+ M3/M0 ratio versus CD4+CXCR5+ICOS+ M3/M0 ratio on an individual basis; each dot illustrates an individual. M0, month 0; M3, month; n=65 for all subfigures.

We analyzed absolute counts of CD4^+^CXCR5^+^ T cells in the blood. These counts showed a heterogeneous evolution over time (**Figures 1A, B**). We categorized the patients into tertiles according to the ratio of CD4^+^CXCR5^+^ T cell counts at month 3 versus at month 0 to further examine the behavior of the Tfh populations (**Figure 1C**). Longitudinal examination of CD4^+^CXCR5^+^ T cells in each group showed different patterns of surface marker expression over time. First, we examined PD1 expression, which is a marker of recent antigen exposure in these cells (31). In patients with the highest expansion of CD4^+^CXCR5^+^ T cells (T3 group), the frequency of PD1^+^CD4^+^CXCR5^+^ cells decreased over time, whereas it increased in those with no expansion (T1 group, **Figure 1D**). Next, we examined the population of CCR7^-^PD1^+^CD4^+^CXCR5^+^ T cells. CCR7 is crucial for recruiting naïve T cells, and with CXCR5, it is essential for secondary lymphoid organ architecture development (32). The CCR7^-^PD1^+^CD4^+^CXCR5^+^ T cells are generated following antigen uptake before the GC, allowing migration to B cells (11). This phenotype confirms active differentiation (31, 33). Consistent with these observations, we noted that at baseline, patients with increased CD4^+^CXCR5^+^ T cell counts had higher percentages of these cells (**Figure 1E**).

Recently activated Tfh cells should express ICOS in addition to PD1 (14, 33). Therefore, we examined ICOS in PD1^+^CD4^+^CXCR5^+^ T cells and observed an expression pattern similar to that of PD1. Patients with the highest CD4^+^CXCR5^+^ progression had a higher proportion of cells co-expressing ICOS and PD1 within this compartment at baseline, which decreased after 3 months (**Figure 1F**). Next, we investigated whether the decrease in PD1 expression correlated with ICOS on a per patient basis. We found a significant relationship between the proportion of PD1 and ICOS expression at 3 months compared with that at baseline (**Figure 1G**). Therefore, patients with increased proportions of circulating CD4^+^CXCR5^+^ T cells showed decreased expression of PD1 and ICOS over time. Collectively, these results suggest that stable kidney recipients who have an expansion of their circulating Tfh cells show a pattern of recent activation at baseline, that attenuates over time.

### CD4^+^CXCR5^+^ T circulating cells expansion correlates with alloreactive CD4^+^ T cells and plasmablasts in the blood and Tfh infiltration in the graft

**Figure 2:**
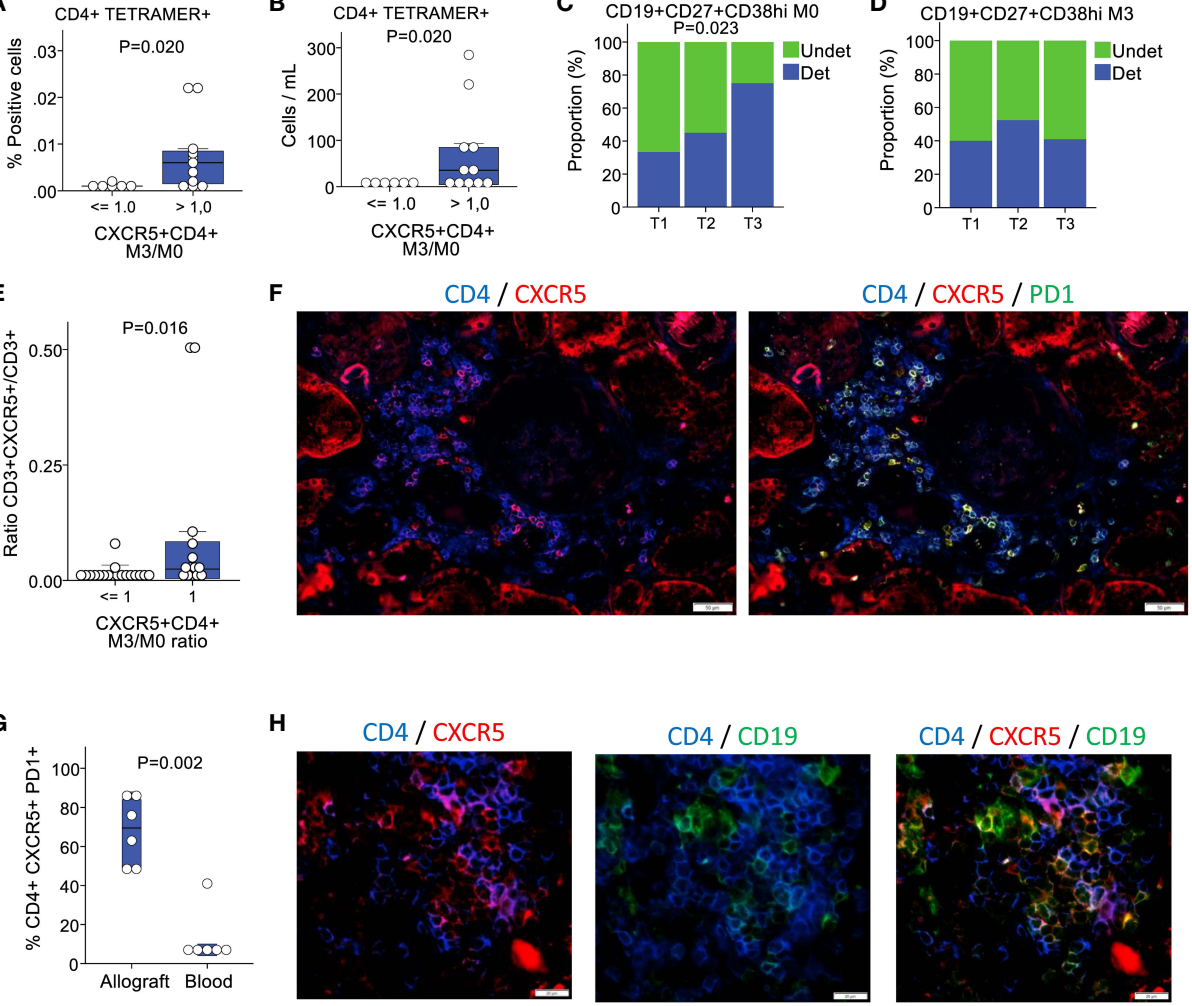
Patients with an expansion of circulating CD4+CXCR5+ T cells present with more circulating HLA-specific alloreactive T cells, plasmablasts and intragraft Tfh cells. **(A)** Proportion of CD4+tetramer+ cells according to the absence or presence of circulating CD4+CXCR5+ T cells increase at 3 months (n=17). **(B)** Corresponding blood cells counts (n=17). **(C)** Baseline proportion of patients with detectable circulating CD19+CD27+CD38hi plasmablasts according to tertiles of circulating CD4+CXCR5+ T cells expansion (n=61). **(D)** Proportion of patients at 3 months for the same cell population (n=63). **(E)** Proportion of CXCR5+ positive cells in the CD3+ compartment in biopsies, according to the absence or presence of circulating CD4+CXCR5+ T cells increase at 3 months (n=32). **(F)** Multicolor immunofluorescence of CD4, CXCR5, and PD1 in a renal allograft. **(G)** Proportion of PD1+ cells in the CD4+CXCR5+ compartment in the allografts versus in the blood n=12). **(H)** Multicolor immunofluorescence of CD4, CXCR5, and CD19 in a renal allograft. M0, month 0; M3, month 3; Undet, undetectable; Det, detectable.

It was recently reported that kidney recipients who develop *de novo* donor-specific HLA antibodies have an enhanced allospecific T-cell response *in vitro*, suggesting an expanded reservoir of donor-specific memory T cells (34). To investigate whether the expansion of CD4^+^CXCR5^+^ T cells was associated with increased allospecific T cells, we used mismatched HLA class II tetramers loaded with nonspecific CLIP. Tetramers unrelated to the donor were used to assess nonspecific binding. These experiments were conducted on 17 patients with donor-recipient mismatches to either one or two HLA-DR1, HLA-DR4, or HLA-DR15 antigens. We compared patients with a ratio of CD4^+^CXCR5^+^ T cells at month 3 versus month 0 that was below or equal to 1 to patients with a ratio above 1. We found tetramer-specific CD4^+^ T cells in 17% (1/6) of the patients with a ratio of ≤1 compared with 73% (8/11) in those with a ratio of >1 (**Figure 2A**). The absolute counts derived from the peripheral blood lymphocyte counts measured for each individual ranged from 0 to 284 cells/mL (**Figure 2B**). Therefore, anti-HLA-specific CD4^+^ T cells were mostly found in patients with an expansion of their pool of CD4^+^CXCR5^+^ T cells at 3 months.

During the immunization process leading to the generation of specific antibodies, the changes occurring in T and B cells culminate in the interaction between the two cell types at the T-B interface (17). However, before this crosstalk occurs, mature B cells originating from marrow precursors circulate in the peripheral blood in search of antigenic stimulation. Following a trivalent influenza vaccine administration to healthy controls, plasmablasts temporarily increase (14). Therefore, we aimed to determine whether there were any repercussions of CD4^+^CXCR5^+^ T cell expansion in circulating plasmablasts. Cells from 61 and 63 patients at months 0 and 3, respectively, were used in this analysis. The proportion of patients with detectable CD19^+^CD27^+^CD38^hi^ plasmablasts in the blood was low at 51% (31/61) and 44% (28/63) at months 0 and 3, respectively. Nearly twice as many patients in the third tertile of CD4^+^CXCR5^+^ T cell expansion had detectable plasmablasts at month 0 compared with the first tertile (**Figure 2C**). No differences were noted at 3 months (**Figure 2D**).

Secondary lymphoid tissues are the main sites of Tfh generation following immunization; however, this process can also occur intragraft in transplant recipients. It is increasingly recognized that extra-follicular responses are the main effector of infections, whereas the GC responses lead to the reshaping of the B cell repertoire with increased adaptation and enhancement of the immune response (11). The currently accepted model is that Tfh cells at the T-B cell border generate both the GC-resident Tfh and circulating CD4^+^CXCR5^+^ T cells (17). In the transplant setting, a complete GC response is prevented by immunosuppressants. Despite immunosuppression, allografts constitute a continuous and inexhaustible reservoir of foreign antigens. Therefore, it is an ideal environment for extrafollicular Tfh cell formation following dendritic cell (DC)-T cell interactions. In mice immunized *in vivo* with ovalbumin, the prolonged presentation of peptides to T cells by DCs resulted in Tfh induction without B cells (35). To determine whether the expansion of circulating CD4+CXCR5+ T cells had correlates in the allotissue, we examined the phenotype of intragraft T cells in biopsies performed during baseline blood sampling. Again, the biopsies showed minimal cell infiltration and did not meet the criteria for rejection. We detected significantly more CXCR5^+^ cells in the CD3^+^ T cell compartment in the biopsies of patients with an expansion of circulating CD4^+^CXCR5^+^ T cells than in those without expansion (**Figure 2E**). Subsequently, we examined CD4, CXCR5, and PD1 expression in patients with circulating CD4^+^CXCR5^+^ T cells expansion (**Figure 2F**). Notably, 68 ± 17% of CD4^+^CXCR5^+^ cells were PD1^+^, which is a proportion substantially higher than in the blood (**Figure 2G**). Additional staining for CD19 showed evidence of direct contact between B cells and CD4^+^CXCR5^+^ T cells in the grafts (**Figure 2H**).

Taken together, these data show that the expansion of the circulating CD4^+^CXCR5^+^ T pool is associated with a higher proportion of allo-immune T cells and plasmablasts in the blood, as well as a higher proportion of CXCR5^+^ cells in the graft, which is mostly PD1^+^ and appears to form T-B contacts *in situ*, similar to a tertiary lymphoid center (36, 37).

### Leucocyte infiltrates in the allograft do not precede the increase in circulating CD4^+^CXCR5^+^ T cells

**Figure 3:**
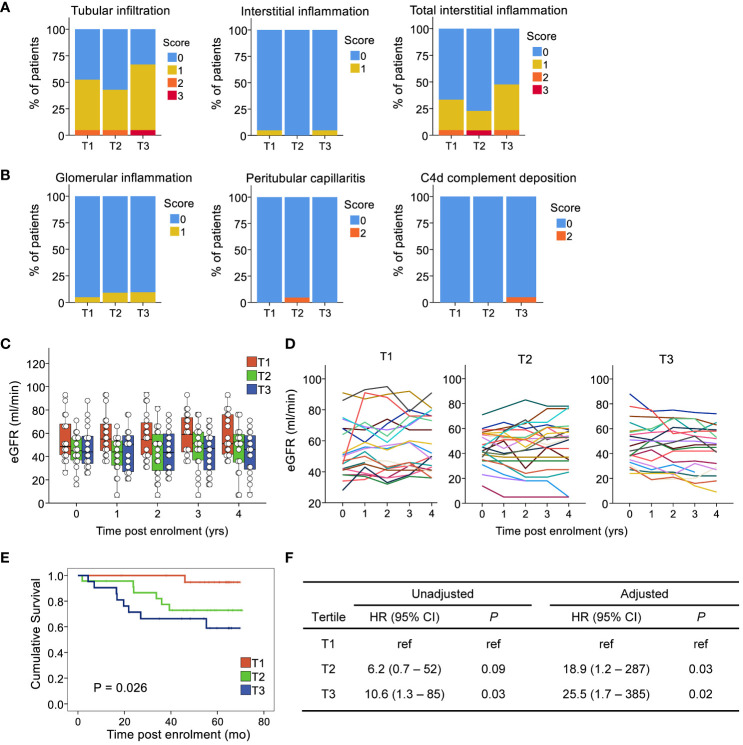
Expansion of circulating CD4+CXCR5+ T cells is not related to prior signs of activation in the allograft, but is associated with poorer clinical outcomes. The compartments assessed for rejection included **(A)** the tubulointerstial compartement: leucocyte infiltration in the tubules in unscared area (tubular infiltration, t score), the interstitium unscared area (interstitial inflammation, i score), the interstitium in both scarred and unscarred area (total interstitial inflammation, ti score) and **(B)** the microvascular compartment: the glomerular capillaries (glomerulitis, g score), and the peritubular capillaries (peritubular capillaritis, ptc score). In addition, the deposition of the C4d component of the complement was assessed (C4d score) as an indirect evidence of antibody ligation to the endothelium in the peritubular capillaries, witnessing upstream C1q activation by antibodies. **(C)** Boxplots and individual values of eGFR post-enrolment. **(D)** Longitudinal eGFR by tertile of circulating CD4+CXCR5+ T cell ratios at 3 months versus baseline; each line illustrates an individual. **(E)** Kaplan-Meier curve of the composite endpoint by tertile. **(F)** Cox models for the composite endpoint, unadjusted and adjusted for recipient age and sex, first transplant, time post transplant, eGFR at first blood sample, and donor type. eGFR, estimated glomerular filtration rate; HR, hazard ratio; n=65 for all subfigures.

Next, we assessed whether individuals who experienced an increase in circulating CD4^+^CXCR5^+^ T cells had evidence of alloreactivity in the graft at baseline according to standard clinical criteria. Notably, we excluded individuals diagnosed with rejection on a baseline biopsy from the study population. Renal pathologists use the standardized Banff International Classification to assess tissue lesions in the different renal compartments (38). Each score ranges from 0 to 3 according to the severity of the lesion. Examination of the mononuclear cells in the tubulointerstitial compartments showed no significant differences between the tertiles (**Figure 3A**). No evidence of endothelial inflammation in the glomerular or peritubular capillaries was found (**Figure 3B**). Additionally, we found minimal deposition of the C4d component, an indirect evidence of antibody ligation to the endothelium in the peritubular capillaries, witnessing upstream C1q activation by antibodies near endothelial cells. Thus, there was no signal for an active rejection process by the current clinical criteria.

### Individuals with an increase in their circulating CD4^+^CXCR5^+^ T cells present with reduced graft function and worse outcomes

We investigated whether an association existed between CD4^+^CXCR5^+^ T cell expansion and subsequent graft function. Except for age, which was lower in the first tertile, the tertiles were not associated with any baseline characteristics such as sex, time post-transplant, donor type, eGFR, or HLA mismatch level (**Table 1**). No association was found with immunosuppressive therapy regarding induction, maintenance agents, or doses. The relationship between each tertile and graft function over time was examined. Calculation of the eGFR up to 4 years after enrollment for each patient revealed a decrease in the third tertile (-4.1 ± 1.7 mL/min versus baseline) but a slight increase in the second (+2.0 ± 2.5 mL/min versus baseline) and first (+4.0 ± 2.3 mL/min versus baseline) (**Figures 3C, D**, P=0.038).

Therefore, to further assess the association between CD4^+^CXCR5^+^ T cell evolution and graft outcomes, we analyzed the development of circulating *de novo* donor-specific anti-HLA antibodies, follow-up biopsies, and survival according to tertiles of CD4^+^CXCR5^+^ T cells in the blood. Overall, during follow-up, 15 patients experienced the composite outcome of *de novo* donor specific anti-HLA antibodies, biopsy-proven rejection, *de novo* decline in the eGFR <30 mL/min, or death-censored graft loss. Among these patients, two had *de novo* donor specific anti-HLA antibodies. The first patient had an anti-B38, with an MFI of 1013 but with epitope 163LW clearly present. The second has an anti-DQ5, with an MFI of 2217. One event occurred in the first tertile, 6 in the second, and 8 in the third. Survival analysis using Kaplan–Meier method and the log-rank test showed a significant difference between tertiles (**Figure 3E**). Proportional hazard modeling indicated that patients in the second and third tertiles were 6 and 11 times more likely to experience the composite outcome, respectively, compared with those in the first tertile (**Figure 3F**). These associations showed no sign of confounding after adjusting for age, sex, first transplant status, renal function at biopsy, and donor type (**Figure 3F**). Such adjustments to demonstrate control of confounding in cohorts with low number of events has been shown to be adequate (39). Therefore, the data indicate that despite the comparable clinical status at baseline, patients with an expansion of their circulating CD4^+^CXCR5^+^ T cells have a poorer prognosis.

## Discussion

The aim of the present study was first to examine the relationship between an expansion of the circulating CD4^+^CXCR5^+^ T cell counts over time, their alloreactive circulating CD4^+^ T cell status, and their correlation with circulating plasmablasts. Additionally, we aimed to determine whether the expansion of CD4^+^CXCR5^+^ T cells was associated with graft infiltration and adverse graft outcomes. We found that the expansion of circulating CD4^+^CXCR5^+^ T cells decreased the proportion of PD1^+^, CCR7^-^PD1^+^, and ICOS^+^ cells among CD4^+^CXCR5^+^ T cells over time. The expansion of CD4^+^CXCR5^+^ T cells correlates with more alloreactive CD4^+^ T cells and plasmablasts in the blood. In the allotissue, we examined the intragraft T cell compartment and found that CD4^+^CXCR5^+^ cells were predominantly PD1-positive and were in direct contact with CD19^+^ B cells. Second, clinically, we found a correlation between Tfh expansion and the composite of long-term decline in graft function, higher incidence of *de novo* anti-HLA donor-specific antibodies, rejection, and graft loss. From an immunological standpoint, these observations suggest that tracking these cells may help detect early alloimmune activation. Therefore, the lack of evidence of clinical rejection and the similar graft function between groups at baseline both indicate that this alloimmune response escapes current surveillance methods, but nonetheless leads to adverse graft outcomes.

The association between circulating Tfh cells pre- and post-transplant, as well as during antibody-mediated rejection, has recently been reported (34, 40–45). Cano-Romero et al. found that patients who developed anti-HLA antibodies following transplantation had an expansion of those cells at 6 months following transplantation. They also observed an association between pre-transplant circulating Tfh levels and acute rejection (40). Similarly, Danger et al. examined circulating Tfh levels on the day of transplant and 1-year post-transplant (46). Contrary to expectations, they reported that a decrease in activated circulating Tfh cells, identified using positive staining for CXCR5, PD1, and ICOS, predicted donor-specific antibody development. To explain this unexpected finding, they speculated that activated CXCR5^+^PD1^+^ICOS^+^ cells might have expanded before the time of blood sampling at 1 year, followed by migration in the tissues and lower levels in the blood. The current study design, which includes the examination of two blood samples collected a few months apart, enabled us to conclude that Tfh expansion over time leads to the attenuation of PD1 and ICOS. Furthermore, the examination of the graft biopsy also supports the hypothesis that CXCR5^+^PD1^+^ cells may either preferentially invade the tissue or develop *in situ*.

From an immunological standpoint, these observations are congruent with the longitudinal Tfh profiles obtained following immunization in a non-transplant setting. Immunization of mice with sheep red blood cells resulted in a transient peak in the number of CCR7^lo^PD1^hi^ cells early after immunization, followed by an increase in the CCR7^hi^PD1^lo^ subset in the lymphoid organs (31). In humans, following influenza vaccination, Bentebibel et al. observed a temporary increase in ICOS expression in CD4^+^ T cells co-expressing CXCR5 and CXCR3 (14). In these two models, the moment of immunization was precisely known, and there was no immunosuppressant that weakened the immune system. In transplant recipients, immunosuppressive drugs severely reduces the immune response. However, the high incidence of chronic, antibody-mediated rejection in the long term is evidence that at some point during the host versus graft response, sufficient T cell priming occurs to trigger the generation of Tfh in a manner similar to vaccination. Recent data showing that alloreactive Tfh cells are primed in allograft-draining nodes suggest that the expansion of Tfh cells in the blood may only represent a proportion of this immune process (47).

In contrast, from a clinical perspective, whether this subtle immune activation of follicular cells is preventable, either by optimization of the current immunosuppression (48) or by new agents targeting the Tfh activation pathway, remains unanswered. First, we found no signal for a different level of immunosuppression between those who increased their circulating Tfh levels and the controls. Second, current knowledge of Tfh physiology suggests that this pathway is potentially amenable to blockade using cytokine-specific agents without affecting all B cells. Anti-IL21 therapy was succesful to treat bronchiolitis obliterans in a mouse model of chronic graft-versus-host disease, whereas depletion of anti-CD20 therapy failed (49). We previously reported that monocyte-derived allogeneic DC polarized naïve T cells into a follicular phenotype, a process that led to a substantial increase in the production of IL-12p40 using a human *in vitro* model (23). Blockade of this p40 subunit decreased the generation of follicular cells in the DC-T cell co-culture. These observations are consistent with previous reports showing that in humans, the cytokine IL-23, composed of p40 and p19 subunits, can replace IL-12 as a Tfh-inducing cytokine (50, 51). Although complete abrogation of the Tfh pathway is probably undesirable in maintaining some immune responsiveness to infectious agents (52), attenuation of its expression might safely achieve the immunosuppressive goal, similar to the effect currently provided by calcineurin inhibitors on general lymphocyte activation and proliferation.

One potential utility of longitudinal tracking of circulating Tfh levels is that their development is probably a slow immunological process in immunosuppressed individuals. Therefore, it is conceivable that serial profiling of a single Tfh marker or a combination of markers tracking several cell subsets could allow timely therapeutic intervention. One argument in favor of this assertion is that the differentiation of naïve T cells into follicular helper cells occurs only through the indirect allorecognition pathway, which is involved in the presentation of HLA antigens and minor histocompatibility antigens, eliciting weaker and slower responses (53). Given the current immunosuppressive load transplant recipients receive, it is of great relevance to personalize Tfh therapeutics to determine for whom and when treatment will be beneficial.

This study had some limitations. First, it was somewhat surprising that in the cohort tested here, few patients in the Tfh expansion group had detectable anti-HLA donor-specific antibodies. The correlation between Tfh levels and hard clinical outcomes supports the notion that the absence of anti-HLA antibodies does not exclude the presence of other non-HLA antibodies that could bind endothelial antigens and trigger tissue damage. Another possibility for the non-detection of anti-HLA antibodies is the episodic and fluctuating nature of their expression in the blood. Indeed, the requirement for detecting donor-specific antibodies to diagnose antibody-mediated rejection was abandoned in 2017 in the Banff classification because of these possibilities (26). Second, the cohort did not include long-term biopsy surveillance to assess histological damage. Nonetheless, a negative clinical outcome associated with Tfh expansion was observed and found to be independent of the usual confounders. The number of cells available limited the characterization of the immune profile. It is known that, within the T follicular cell compartment, subsets differentially support antibody secretion, and - CD8^+^CXCR5^+^ T cells may be associated with reduced donor-specific alloantibody production (20, 54–56). The low number of patients who received ATG in the cohort limited the capacity to determine whether depletional induction therapy had an impact on the associations studied.

In summary, the results reported here further support the notion that tracking circulating Tfh levels may be useful in the clinic for detecting early immune activation in transplant recipients since it correlates with increased CD4^+^ alloreactivity, plasmablast counts, Tfh graft infiltration, and worse clinical outcomes. Therefore, these data provide an impetus to better understanding how Tfh activation can be safely limited in these populations.

## Data availability statement

The raw data supporting the conclusions of this article will be made available by the authors, without undue reservation.

## Ethics statement

The studies involving humans were approved by University Health Center (CHU) of Quebec Ethics Committee, project 2017-3606-F9-54172. The studies were conducted in accordance with the local legislation and institutional requirements. The participants provided their written informed consent to participate in this study.

## Author contributions

OD: Conceptualization, Investigation, Writing – original draft, Writing – review & editing. SB: Funding acquisition, Investigation, Writing – review & editing. MP-T: Investigation, Writing – review & editing. MM: Investigation, Writing – review & editing. J-SD: Investigation, Writing – review & editing. FB-B: Funding acquisition, Writing – review & editing. AG: Writing – review & editing. JR: Investigation, Writing – review & editing. EL: Investigation, Writing – review & editing. SS: Conceptualization, Funding acquisition, Investigation, Supervision, Writing – original draft, Writing – review & editing.

## Supplementary material

Supplementary Figure 1 Gating strategy.
